# Effects of BRCA2 cis-regulation in normal breast and cancer risk amongst BRCA2 mutation carriers

**DOI:** 10.1186/bcr3169

**Published:** 2012-04-18

**Authors:** Ana-Teresa Maia, Antonis C Antoniou, Martin O'Reilly, Shamith Samarajiwa, Mark Dunning, Christiana Kartsonaki, Suet-Feung Chin, Christina N Curtis, Lesley McGuffog, Susan M Domchek, EMBRACE, Douglas F Easton, Susan Peock, Debra Frost, D Gareth Evans, Ros Eeles, Louise Izatt, Julian Adlard, Diana Eccles, GEMO Study Collaborators, Olga M Sinilnikova, Sylvie Mazoyer, Dominique Stoppa-Lyonnet, Marion Gauthier-Villars, Laurence Faivre, Laurence Venat-Bouvet, Capucine Delnatte, Heli Nevanlinna, Fergus J Couch, Andrew K Godwin, Maria Adelaide Caligo, SWE-BRCA, Rosa B Barkardottir, kConFab Investigators, Xiaoqing Chen, Jonathan Beesley, Sue Healey, Carlos Caldas, Georgia Chenevix-Trench, Bruce AJ Ponder

**Affiliations:** 1 Cambridge Research Institute - CRUK, Li Ka Shing Centre, Cancer Research UK, Robinson Way, Cambridge, CB2 0RE, UK; 2 Department of Oncology, University of Cambridge, Addenbrooke's Hospital, Robinson Way, Cambridge, CB2 0RE, UK; 3 Centre for Cancer Genetic Epidemiology, Department of Public Health and Primary Care, University of Cambridge, Worts Causeway, Cambridge CB1 8RN, UK; 4 Department of Medicine, Hematology-Oncology, Abramson Cancer Center, University of Pennsylvania, 3400 Spruce St, Philadelphia, PA 19104, USA; 5 Genetic Medicine, Manchester Academic Health Sciences Centre, Central Manchester University Hospitals NHS Foundation Trust, Brunswick Street, Manchester, M13 9PL, UK; 6 Oncogenetics Team, The Institute of Cancer Research and Royal Marsden NHS Foundation Trust, 15 Cotswold Rd, Belmont, Sutton Surrey SM2 5NG, UK; 7 Clinical Genetics, Guy's and St. Thomas' NHS Foundation Trust, 7th floor, Borough Wing, Guy's Hospital, Great Maze Pond, London SE1 9RT, UK; 8 Yorkshire Regional Genetics Service, Ward 10, 3rd Floor, Chapel Allerton Hospital Chapeltown Road, Leeds, LS7 4SA, UK; 9 Wessex Clinical Genetics Service, Princess Anne Hospital, Coxford Road, Southampton, SO16 5YA, UK; 10 GEMO Study Collaborators: Cancer Genetics Network "Groupe Génétique et Cancer", Fédération Nationale des Centres de Lutte Contre le Cancer, France; 11 INSERM U1052, CNRS UMR5286, Université Lyon 1, Cancer Research Center of Lyon, Lyon, 7 rue Guillaume Paradin, 69008 Lyon, France; 12 Unité Mixte de Génétique Constitutionnelle des Cancers Fréquents, Centre Hospitalier Universitaire de Lyon/Centre Léon Bérard, 28 rue Laennec, 69008 Lyon, France; 13 Service de Génétique Oncologique, Institut Curie, 26 rue d'Ulm 75248 Paris cedex 05, France; 14 Unité INSERM U830, Institut Curie, 26 rue d'Ulm 75248 Paris cedex 05, France; 15 Université Paris Descartes, Faculté de Médecine, 12, rue de l'Ecole de Médecine 75270 Paris Cedex 06, France; 16 Centre de Génétique, CHU Dijon, Université de Bourgogne, Dijon F-21000, France; 17 Centre Georges François Leclerc, 1 Rue Professeur Marion 21000 Dijon, France; 18 Department of Medical Oncology, Centre Hospitalier Universitaire Dupuytren, Limoges, France; 19 Centre René Gauducheau, Boulevard Jacques Monod 44805 St Herblain Cedex, Nantes, France; 20 Department of Obstetrics and Gynecology, Helsinki University Central Hospital, P.O. BOX 700, 00029 HUS, Finland; 21 Department of Laboratory Medicine and Pathology, Mayo Clinic, 200 1st Street Southwest Rochester, MN 55905, USA; 22 University of Kansas Medical Center, 3901 Rainbow Boulevard, KS City, KS 66160, USA; 23 Division of Surgical, Molecular and Ultrastructural Pathology, Department of Oncology, University of Pisa and Pisa University Hospital, Lungarno Antonio Pacinotti, 43 56126 Pisa, Italy; 24 Department of Pathology, Landspitali University Hospital, Reykjavik 101, Iceland; 25 Faculty of Medicine, University of Iceland, Vatnsmýrarvegur 16, level 4 Reykjavik, Iceland; 26 Peter MacCallum Cancer Institute, Locked Bag 1, A'Beckett Street, Melbourne, VIC 8006, Australia; 27 Queensland Institute of Medical Research, 300 Herston Road, Herston, Brisbane, QLD 4006, Australia; 28 Cambridge Experimental Cancer Medicine Centre, Li Ka Shing Centre, Robinson Way, Cambridge, CB2 0RE, UK; 29 Institute for Biotechnology and Bioengineering, Centre for Molecular and Structural Biomedicine, Department of Biomedical Sciences and Medicine, University of Algarve, Portugal; 30 Institute for Biotechnology and Bioengineering, Centre for Molecular and Structural Biomedicine, Department of Biomedical Sciences and Medicine, Gambelas Campus, Building 7, University of Algarve, 8005-139 Faro, Portugal

## Abstract

**Introduction:**

*Cis*-acting regulatory single nucleotide polymorphisms (SNPs) at specific loci may modulate penetrance of germline mutations at the same loci by introducing different levels of expression of the wild-type allele. We have previously reported that *BRCA2 *shows differential allelic expression and we hypothesize that the known variable penetrance of *BRCA2 *mutations might be associated with this mechanism.

**Methods:**

We combined haplotype analysis and differential allelic expression of *BRCA2 *in breast tissue to identify expression haplotypes and candidate cis-regulatory variants. These candidate variants underwent selection based on *in silico *predictions for regulatory potential and disruption of transcription factor binding, and were functionally analyzed *in vitro *and *in vivo *in normal and breast cancer cell lines. SNPs tagging the expression haplotypes were correlated with the total expression of several genes in breast tissue measured by Taqman and microarray technologies. The effect of the expression haplotypes on breast cancer risk in *BRCA2 *mutation carriers was investigated in 2,754 carriers.

**Results:**

We identified common haplotypes associated with differences in the levels of *BRCA2 *expression in human breast cells. We characterized three *cis*-regulatory SNPs located at the promoter and two intronic regulatory elements which affect the binding of the transcription factors C/EBPα, HMGA1, D-binding protein (DBP) and ZF5. We showed that the expression haplotypes also correlated with changes in the expression of other genes in normal breast. Furthermore, there was suggestive evidence that the minor allele of SNP rs4942440, which is associated with higher *BRCA2 *expression, is also associated with a reduced risk of breast cancer (per-allele hazard ratio (HR) = 0.85, 95% confidence interval (CI) = 0.72 to 1.00, *P*-trend = 0.048).

**Conclusions:**

Our work provides further insights into the role of *cis*-regulatory variation in the penetrance of disease-causing mutations. We identified small-effect genetic variants associated with allelic expression differences in *BRCA2 *which could possibly affect the risk in mutation carriers through altering expression levels of the wild-type allele.

## Introduction

Unequal expression of the alleles of autosomal genes is common in the human genome [1,2]. This differential allelic expression (DAE) is thought to play a major role in intra-species phenotypic variation as well as individual susceptibility to complex diseases. Previous reports have suggested a role for DAE in cancer, including that of the colon [3,4], the pancreas [5], the ovary [6] and the breast [7,8]. Additionally, several cancer risk-associated SNPs identified through genome-wide association studies (GWAS) have been shown to have a role in gene expression regulation [9-13]. We previously reported that *BRCA2 *displays DAE in breast and blood samples from healthy individuals [14]. Using a coding single nucleotide polymorphism (SNP) in exon 9 of *BRCA2*, rs144848, as a marker for quantifying allelic transcripts in heterozygous individuals, we observed differences between alleles as large as four-fold. Additionally, our data suggested that the *cis*-regulatory variation responsible for DAE is in the same haplotype block as rs144848.

Deleterious germline mutations in *BRCA2 *are rare but confer a high risk of breast cancer. However, penetrance estimates vary and several lines of evidence indicate that other genetic and environmental factors modify the cancer risks conferred by these mutations [15-17]. Studies by the Consortium of Investigators of Modifiers of *BRCA1/2 *(CIMBA) have demonstrated that common alleles in *RAD51, FGFR2, MAP3K1, TOX3/TNRC9, LSP1, SLC4A7/NEK10*, a 2q35 locus and a 5p12 locus affect breast cancer risk in *BRCA2 *mutation carriers [18-20]. These loci account for approximately 4% of the genetic variation observed in the penetrance of *BRCA2 *mutations in breast cancer.

Most of the deleterious germline mutations in *BRCA2 *introduce premature termination codons and trigger nonsense-mediated mRNA decay [11]. Haploinsufficiency in *BRCA2^+/- ^*cells has been shown to affect DNA damage repair of breaks induced by γ-irradiation and mitomycin C [21] which can contribute to cancer predisposition in mutation carrier families. Recently, it was also reported that primary breast epithelial cells cultured from *BRCA2 *mutation carriers and controls have differences in gene expression signatures [22].

In this study, we set out to identify and characterize *cis*-regulatory variation responsible for DAE in *BRCA2 *and to determine whether the penetrance of *BRCA2 *mutations could be modified by the variable level of expression of the remaining wild-type allele in heterozygous mutation carriers. We identified common haplotypes that associate with different levels of *BRCA2 *expression and found three SNPs that alter the binding affinity of transcription factors and overlap *cis*-regulatory elements. The expression haplotypes were also found to correlate with altered expression in breast tissue of genes whose expression has previously been reported to be altered in the presence of inherited *BRCA2 *mutations. We found one haplotype that was associated with higher expression of *BRCA2*, for which there is suggestive evidence that it is also associated with a protective effect for the development of breast cancer in *BRCA2 *mutation carriers.

## Methods

### SNP and haplotype analysis

Hapmap release 16 and data from the 1,000 Genomes Project were accessed [23-26]. Haplotype structure and linkage disequilibrium analysis were performed using Haploview software [27,28].

### Human tissue samples

B cells and normal breast tissue from healthy individuals and normal matched breast tissue from cancer patients were obtained from the Blood Centre and the Tissue Bank at Addenbrooke's Hospital as previously described [14], with approval from the Addenbrooke's Hospital Local Research Ethics Committee (REC reference 04/Q0108/21, 06/Q0108/221 and 07/H0308/161, respectively). The B cell and control normal breast tissue material was used for the functional analysis and the DAE study. The normal matched breast tissue was used for total gene expression studies.

### Nucleic acid extraction and processing

DNA was extracted using a conventional SDS/proteinase K/phenol method. Total RNA was extracted from all samples using Qiazol (QIAGEN, Crawley, UK) following the manufacturer's instructions. The RNA was subsequently treated with DNaseI and repurified using acidic phenol-chloroform, and ethanol precipitation. DNA and RNA integrity were checked using the Agilent Bioanalyzer (Agilent Technologies, Santa Clara, CA, USA).

cDNA was prepared from total RNA with the SuperScript^® ^III First Strand SuperMix (Invitrogen, Carlsbad, CA, USA) using random hexamers and oligo-dT primers, according to the manufacturer's instructions.

### Breast cell lines culture

Breast cancer (PMC42, MCF-7 and SUM-159) and normal breast (MCF-10A) cell lines were cultured as previously described [29-32] for extracting nuclear protein for electrophoretic mobility shift assays and for extracting chromatin for immunoprecipitation assays.

### Genotyping of normal blood and breast tissue from healthy individuals

Genotyping for rs144848 was performed using a fluorescent 5' exonuclease TaqMan assay and the ABI PRISM 7900 Sequence Detection Sequence (Applied Biosystems, Foster City, CA, USA). Genotyping for another six SNPs that tag common haplotypes for the DAE region in *BRCA2 *was carried out by PCR amplification of 150 bp to 250 bp around the SNP of interest. Primer sequences are provided in the Supplementary Material (Additional file 1 Table S1). Products were verified by agarose gel electrophoresis and sequenced using ABI Big Dye chemistry and capillary electrophoresis on an ABI 3730 sequencer (Applied Biosystems).

### Affymetrix Genotyping

Thirty-seven normal breast tissue samples were genotyped using Affymetrix Genome-Wide SNP 6.0 arrays (Affymetrix, Santa Clara, CA, USA), following the standard Affymetrix protocol using 0.5 μg DNA, at AROS Applied Biotechnology (Aarhus, Denmark). Affymetrix SNP 6.0 arrays data were pre-processed and analyzed using the crlmm Bioconductor package [33] implemented in the R statistical programming language [34] (GEO: GSE32259).

### Quantification of differential allelic gene expression

Differential allelic expression assays were performed as previously described [14]. Validation was performed by Sanger sequencing. In brief, replicate PCRs were performed amplifying 100 bp to 200 bp around the coding SNP of interest; products were verified by agarose gel electrophoresis and sequenced using ABI Big Dye chemistry and capillary electrophoresis on an ABI 3730 sequencer (Applied Biosystems). Allelic expression ratios in cDNA samples were normalized against the genomic DNA ratios. Primer sequences are provided as Supplementary Material (Additional file 1 Table S1).

### *In silico *prediction of transcription factor binding

TRANSFAC professional database [35] (Biobase, Germany) ver.2009.4 with vertebrate non redundant matrices, and MATCH algorithm with core match and matrix match set to minimize the sum of both false positives and negative motif matches were used to identify putative transcription factor binding sites.

### Electrophoretic mobility shift assay (EMSA)

EMSA were performed as previously described [9]. Each EMSA was repeated two or more times for all combinations of cell extract and oligonucleotide, which were also tested in serial diluting amounts. Oligonucleotide sequences are provided as Supplementary Material (Additional file 1 Table S1). Antibodies used for super-shift competition assays are listed in the Chromatin Immunoprecipitation section.

### Chromatin Immunoprecipitation (ChIP)

ChIP experiments were performed using chromatin extracted from SUM-159 (cancer), PMC42 (cancer), MCF-7 (cancer) and MCF10-A (normal) breast cell lines with antibodies against RNA polymerase II (Abcam, Cambridge, UK, ab5408-100), H3K79me2 (Abcam, ab3594), HMGA1a/HMGA1b (Abcam, ab4078), DABP (Insight Biotechnology Ltd, Wembley, UK, sc-98411-X), ZFP161 (Abcam, ab68116), C/EBPα (Insight Biotechnology Ltd, sc-9314-X), IgG-Rabbit (Insight Biotechnology Ltd, sc-2027) and IgG Mouse (Insight Biotechnology Ltd, sc-2025) as recently described [36]. The immunoprecipitated material was purified using QIAquick PCR Cleaning kit (Qiagen) and analyzed by PCR for the regions of interest. Primer sequences are supplied in the Supplementary Material (Additional file 1 Table S1).

### Gene Expression Analysis

The relative gene expression of *BRCA2, SPP1, MUC16, PAX8, BIRC5 *and *RRM2 *was analyzed using Fluidigm 96.96 Dynamic Arrays (Fluidigm, San Francisco, CA, USA), according to the manufacturer's instructions, in a set of normal breast tissue from healthy donors (n = 58). Four housekeeping genes (*Actinβ, HPRT1, 18S *and *GAPDH*) were included in the experiments for normalization purposes.

In short, 1.25 μL of cDNA was pre-amplified in a 5 μL reaction for the specific targets (0.2x of each assay), for eight cycles. Pre-amplified samples were diluted 1:5 before being utilized. Chips were run on the BioMark™ Real-Time PCR System (Fluidigm). The cycling program used consisted of 10 minutes at 95°C followed by 40 cycles of 95°C for 15 seconds and one minute at 60°C. Data were analyzed using the BioMark Gene Expression Data Analysis software to obtain Ct values. Each chip (experiment) was run with all assays plated in triplicate and 94 test samples plus two no-template control samples. Analysis was performed using the ΔΔCt method and normalization was done using the average Ct value for the two housekeeping genes (*Actinβ *and *HPRT1*) that had the most similar distribution of Ct values across arrays.

Additionally, we analyzed separately 33 normal breast tissue samples from healthy individuals and 200 normal breast tissue samples from cancer patients (normal-matched) without *BRCA2 *germline mutations, using Illumina HT12 v3 expression arrays (Illumina, San Diego, CA, USA) (GEO: GSE32259 and EGA: EGAS00000000082). Briefly, for each sample set the bead array package [37] was employed to pre-process and summarize each Illumina H12 v3 BeadChip following quality assessment and the adjustment of spatial artefacts with the BASH tool [38]. Potential outlier arrays were removed by considering the bead-level quality assurance (QA) scores derived using the control probes on each array and the remaining samples were quantile normalized.

### Gene expression statistical analysis

All statistical analysis was performed using R version 2.10.1 [34], unless otherwise stated. Differential allelic expression data were analyzed as previously described [14]. Comparison of proportions between groups for haplotype correlation with DAE was tested using the Chi-square test. The non-parametric Wilcoxon rank sum test was used for the analysis of *BRCA2 *expression versus haplotype. Specific SNP genotype-gene expression correlations were determined using analysis of variance (ANOVA) testing for the first type of correlation and the Student's t test for the second.

### Associations with breast cancer risk for BRCA2 mutation carriers

Associations with breast cancer risk were evaluated using women with *BRCA2 *mutations from 11 studies from the CIMBA consortium for the seven SNPs defining the gene expression associated haplotypes. Subjects participated in clinical or research studies at the host institutions under ethically approved protocols and written informed consent was obtained (Additional file 2 Table S2).

Studies included per SNP in our analysis are shown in Additional file 3 (Table S3). Sample eligibility and available information were reported in detail previously and are provided in Additional file 4[39]. The DNA samples of ten studies were genotyped by iPLEX Mass Array platform (Sequenom Inc., Newton, MA, USA) at Queensland Institute of Medical Research, with a further study, GEMO, genotyped elsewhere by Taqman assay, using Applied Biosystems reagents (Applied Biosystems), for the same seven SNPs. Further details on the genotyping and quality control are described in Additional file 4.

Details of the statistical analysis methods for evaluating the associations between SNP genotypes and breast cancer risk have been published previously and are provided in Additional file 4[18]. In short, we tested the frequency of each SNP in affected (cases) and unaffected (control) *BRCA2 *germline mutation carriers. The effect of each SNP was modelled either as a per-allele HR (multiplicative model) or as separate HRs for heterozygotes and homozygotes, and these were estimated on the log scale. Analyses were carried out with the pedigree-analysis software MENDEL [40]. Heterogeneity between studies was tested by comparing the models that allowed for study-specific log-HRs against models in which the same log-HR was assumed to apply to all studies.

Disease associations for the five common expression haplotypes were also explored using the software HAPSTAT [41], under a standard cohort analysis model and using the low expression haplotype 2 as the reference. It should be noted that this analysis does not account for the sampling design and familial relationships of the participating studies.

## Results

### Haplotype correlation with levels of expression

Recently, we reported differential allelic expression of *BRCA2 *in both normal breast and blood tissue samples from control individuals [14]. Using the coding SNP rs144848 for allelic transcript quantification and an allelic expression ratio threshold of 1.2 (log_2 _= 0.263), we found that in 45 heterozygous samples tested 29 (64%) significantly expressed more of the G allele. All heterozygotes showing DAE preferentially expressed the same allele, indicating that the coding SNP rs144848 is in linkage disequilibrium (LD) with the regulatory variant(s). Linkage disequilibrium is however not complete between the coding and the regulatory SNP(s) as suggested by the fraction of heterozygotes that did not show DAE, who are likely homozygous for the regulatory variant(s).

**Figure 1:**
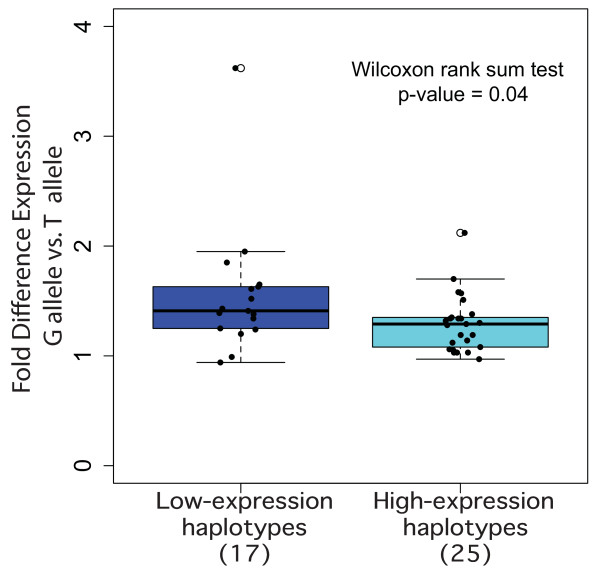
**Differential allelic expression association with ***BRCA2 ***haplotypes in blood and breast**. Fold difference between the G and the T allele for samples heterozygous for rs144848. 'Low-Expression Haplotypes' are significantly associated with differential allelic expression and correspond to haplotypes 2 and 5, and 'High-Expression Haplotypes are not associated with differential allelic expression and correspond to haplotypes 3 and 4.

In this study, for the identification of possible *cis*-regulatory polymorphisms we first analyzed the region surrounding *BRCA2*, which shows very complex linkage disequilibrium. Using data from HapMap (Data release 24/phase II, dbSNP build 126) and the Haploview software, we identified three main haplotype blocks with two further regions without clear block structure, corresponding to five common haplotypes in the European population (CEU samples in HapMap) (tag SNPs for the haplotypes are shown in Table 1). The G allele of the coding SNP rs144848 tags one of these haplotypes, haplotype 1. As the samples used for the DAE study are all heterozygous for rs144848, all samples carry haplotype 1. To evaluate the association between the other haplotypes and the observed DAE we determined the second haplotype in 41 samples heterozygous for rs144848 used in the DAE study. For this we genotyped the samples for the six tag SNPs defining haplotypes 2/3/4/5 and correlated them with the presence or absence of DAE ('DAE' versus 'no DAE' in Table 1). All samples carrying haplotype 2 showed DAE (Sign Test *P*-value = 0.031) and samples with haplotype 5 showed a non-significant predominance in the DAE group. Haplotypes 3 and 4 were found in equal frequency in individuals with and without DAE. Correlation of DAE ratios versus haplotype shows (Figure 1) that haplotypes 2 and 5 have, on average, a larger difference in the allelic expression than haplotypes 3 and 4, meaning that the T allele of rs144848 is less expressed than the G allele when it is inherited as haplotype 2 or 5. In view of these results we defined haplotypes 2 and 5 as 'lower expression' and haplotypes 1, 3 and 4 as 'higher expression' haplotypes.

**Table 1:** Haplotype frequencies in BRCA2, tag SNPs and DAE distribution

HAPLOTYPE PATTERN	Freq (%) HapMap CEU	rs1799943	rs11571579	rs9534174	rs206070	rs144848	rs4942440	rs9567576	DAE	No DAE
hap1	27.5	G	T	**A**	C	**G**	G	T	29	12
hap2	24.2	**A**	**C**	G	C	T	G	**G**	10*	0*
hap3	18.8	G	T	**A**	**T**	T	G	T	4	4
hap4	11.7	G	T	G	C	T	**A**	T	10	7
hap5	5	G	**C**	G	C	T	G	**G**	5	1

* *P*-value = 0.031, Sign-Test. CEU, Utah residents with Northern and Western European ancestry from the CEPH collection; DAE, differential allelic expression; SNP, single nucleotide polymorphism.

### Gene expression profile of breast tissue is dependent on haplotype

**Figure 2:**
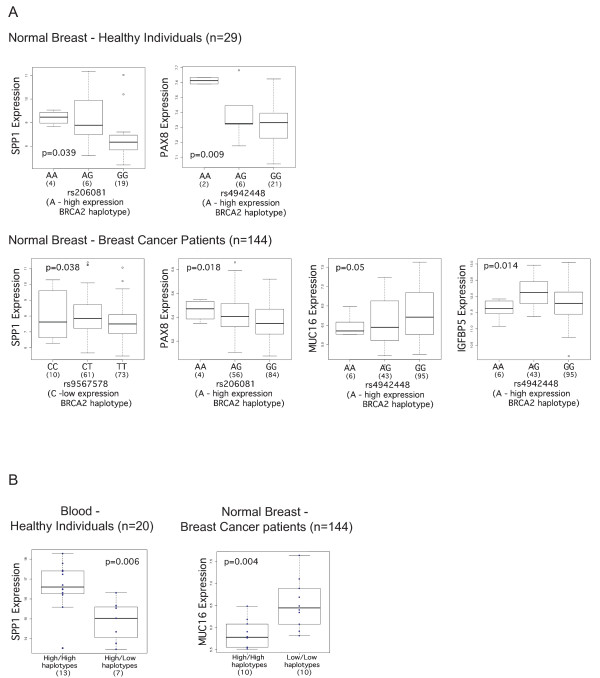
**Differential gene expression analysis in normal breast tissue (controls and breast cancer patients) and normal blood samples**. Box plots displaying differentially expressed genes in normal breast samples from healthy controls (n = 29), matched normal breast tissue from breast cancer patients (n = 144) and blood samples from healthy individuals (n = 20) analyzed by Illumina HT12 v3 arrays and real-time PCR. (**A**) Gene expression versus genotype correlations between the genes *SPP1, PAX8, MUC16 *and *IGFBP5 *and two SNPs associated with higher expression of *BRCA2*, rs206081 (haplotype 3) and rs4942448 (haplotype 4), and one SNP associated with lower expression of *BRCA2*, rs9567578 (haplotype 2); *P*-values correspond to ANOVA testing. (**B**) Gene expression versus expression haplotype correlations for the genes *MUC16 *and *SPP1*; **P**-values correspond to a two-sided Student's t-Test. ANOVA, analysis of variance; PCR, polymerase chain reaction; SNP, single nucleotide polymorphism.

Having identified different expression haplotypes for *BRCA2 *we investigated whether these had an effect on the expression of the eleven genes reported to be altered by Bellacosa and colleagues [22] in *BRCA2 *mutation carriers. We measured the expression level of *IGFBP5, SPP1, RRM2, BIRC5, MUC16 *and *PAX8 *by quantitative real-time PCR and of all eleven genes using Illumina HT12 v3 expression microarrays in the normal breast tissue of healthy controls, in the normal breast tissue of breast cancer patients (non-carriers of *BRCA2 *germline mutations) and also in blood from healthy controls. We analyzed the results in two ways. The first consisted of correlating gene expression versus genotype at specific SNPs that tag different haplotypes, using a linear regression model and ANOVA analysis. In this analysis the heterozygous and the common homozygous groups consist of a mixture of high and low expression haplotypes. To reduce this noise in the data, in the second type of analysis we assigned samples to three groups: individuals carrying two high expression haplotypes, individuals carrying one high and one low expression haplotype, and individuals carrying two low expression haplotypes. We performed Student's t test analysis for the expression of each gene between each two groups. This approach implied a reduction in the number of informative samples. We found evidence of correlation between four out of eleven genes (*SPP1, PAX8, MUC16 *and *IGFBP5*) and the expression haplotypes (Figure 2) which we describe in detail below.

In normal breast tissue from healthy individuals, higher expression of *SPP1 *was found in cells homozygous for the rare allele of rs206081, an SNP in strong LD with the tag for the high-expression haplotype 3 (*P*-value = 0.039, Figure 2A). This correlation was in concordance with the expression haplotype analysis in blood of healthy individuals using real-time PCR data, in which expression of *SPP1 *was found to be significantly higher in individuals carrying two high expression haplotypes (*P*-value = 0.006, difference expression = 1.74, Figure 2B), when compared with individuals carrying one high and one low expression haplotype (no individuals were identified with two low expression haplotypes in these samples). This correlation was found to be in the opposite direction in the normal tissue of breast cancer patients, with lower *SPP1 *expression associated with a low *BRCA2 *expression haplotype (*P*-value = 0.038, Figure 2A). The gene *PAX8 *showed a concordant correlation in normal breast from healthy controls and cancer patients, showing upregulation in rare homozygous samples for SNPs that correspond to haplotypes 3 (rs206081) and 4 (rs4942448), both associated with higher expression of *BRCA2 *(*P*-value = 0.009 in healthy individuals, *P*-value = 0.018 in cancer patients; Figure 2A). The expression of *MUC16 *in the normal tissue of patients was negatively correlated with the A allele of rs4942448 (haplotype 4) (*P*-value = 0.05 in Figure 2A). The upregulation of *MUC16 *in individuals carrying two low-expression haplotypes was confirmed with the second analysis method (*P*-values = 0.004 in Figure 2B). The difference in expression between the high-expression and low-expression haplotype groups was -0.125 for the two probes. In the normal tissue of patients, the expression of *IGFBP5 *was significantly lower in samples homozygous for rs4942448 (haplotype 4), which is associated with lower *BRCA2 *expression (*P*-values = 0.014, Figure 2A).

These results suggest that even small differences in the expression level of *BRCA2*, as those associated with the different haplotypes that we identified, can have an impact on the gene expression profile of normal breast tissue in individuals carrying two copies of *BRCA2*.

### Associations with breast cancer risk for *BRCA2 *mutation carriers

The seven SNPs that characterize the five expression haplotypes identified in the DAE study were genotyped in 2,754 *BRCA2 *mutation carriers from 11 centers from the CIMBA consortium (Additional file 3 Table S3, Additional file 5 Table S4). In total, 1,617 carriers were censored at a first breast cancer diagnosis and 1,137 were treated as unaffected in the analysis. One SNP displayed a significant association with breast cancer in carriers of *BRCA2 *mutations (Table 2) at the 5% significance level. The minor allele of SNP rs4942440, tagging the high expression haplotype 4, was associated with a reduced breast cancer risk (per-allele HR = 0.85, 95% C.I. = 0.72 to 1.00, *P*-trend = 0.048). There was no evidence of heterogeneity in the HRs across studies (*P*-heterogeneity = 0.20, Additional file 6 Figure S1). No associations were observed with the other SNPs (Additional file 5 Table S4). Similar to the gene expression analysis presented above, the association analysis based on each SNP in isolation does not describe the effects of each haplotype separately, as there is no distinction between low and high expression haplotypes in the heterozygous and common homozygous groups.

**Table 2:** rs4942440 genotype frequencies in BRCA2 mutation carriers by disease status and hazard-ratio estimate

	Unaffected (%)	Affected (%)	HR	95% CI	*P *Value
GG	648 (70.36)	817 (73.47)	1		
AG	246 (26.71)	271 (24.37)	0.84	(0.70, 1.01)	
AA	27 (2.93)	24 (2.16)	0.76	(0.43, 1.36)	
2-df test					0.13
Per allele			0.85	(0.72, 1.00)	0.048

CI, confidence interval; HR, hazard ratio.

To address this fact we performed a haplotype analysis using standard cohort analysis in which the low-expression haplotypes 2 and 5 were considered as reference. This revealed that each copy of the higher-expression haplotype 4 was associated with a reduced risk of breast cancer for *BRCA2 *mutation carriers (HR = 0.84, 95%CI: 0.73 to 0.97, *P *= 0.014) (Additional file 7 Table S5). However, this analysis does not take into account the non-random sampling of mutation carriers with respect to disease phenotype, or possible family relations within the individuals studied, so inference based on this analysis is not directly comparable with the single SNP analysis which was based on modelling the retrospective likelihood. Nevertheless, this is also suggestive of an association between a higher expression haplotype of *BRCA2 *in mutation carriers with lower risk of developing breast cancer.

### Identification of regulatory variation

**Figure 3:**
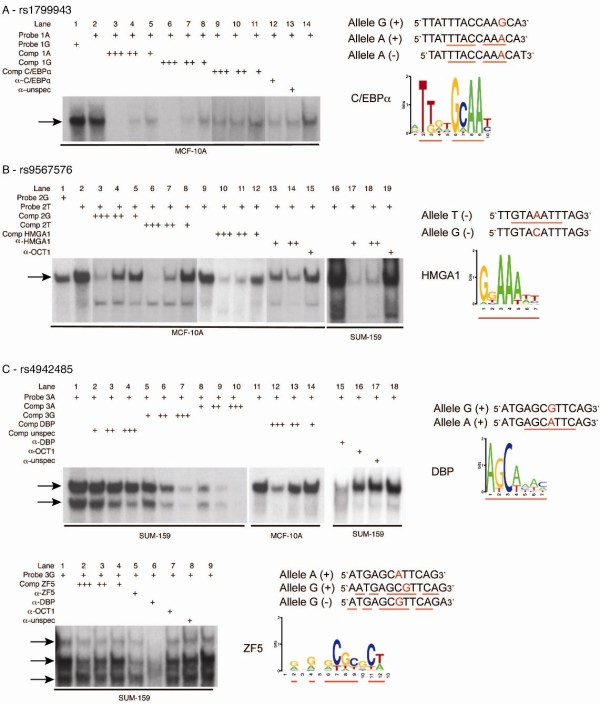
***In silico *and *in vitro *DNA-protein binding studies**. EMSA results, binding sequence motifs for each transcription factor and the DNA sequence around each SNP (alleles in red) are shown. Sequences are shown in the strand direction for which the prediction was found, and red underlines indicate homology between TF motif and allele sequence. DNA motifs were obtained from TRANSFAC and Schmidt *et al*. (C/EBPα) [12,50]. Arrows indicate specific bands for each EMSA. (**A**) Both alleles of rs1799943 show weak *in vitro *binding of C/EBPα. (**B**) Analysis of rs9567576, using cell extract from two cell lines as indicated, and competition assays indicate stronger binding of HMGA1 to the probe containing the T allele. (**C**) EMSA analysis of rs4942485 for the A allele (upper panel) and G allele (lower panel). The A allele shows strong binding to DBP, as identified by competition assays. When using the probe containing the G allele, a lower third band appears in the presence of Zn^2+ ^in the buffer, identified as binding to ZF5 in competition assays. EMSA, electrophoretic mobility shift assay; DBP, d-binding protein; SNP, single nucleotide polymorphism; TF, transcription factors.

**Figure 4:**
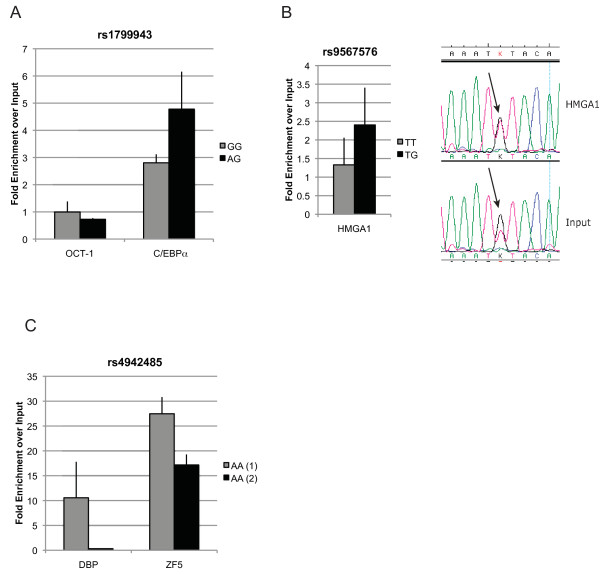
***In vivo *analysis of transcription factor occupancy at *cis*-regulatory sites**. The data show occupancy fold enrichment compared to chromatin input, and corrected against a negative control. The genotype of the cell lines used for each experiment is shown in the legend. (**A**) PCR analysis for ChIP of OCT-1 and C/EBPα at the rs1799943 site. (**B**) PCR analysis for ChIP of HMGA1 at the rs9567576 site. Sequencing chromatograms showing preferential pull-down of T allele in the immunoprecipitate in comparison with input chromatin in a heterozygous cell line. (**C**) PCR analysis for ChIP of DBP and ZF5 at the rs4942485 site. The data are the average of three replicates ± SD in two different cell lines. ChIP, chromatin immunoprecipitation; DBP, d-binding protein; PCR, polymerase chain reaction; SD, standard deviation.

Next we aimed to dissect the genetic *cis*-regulation responsible for the haplotype differences in the expression level of *BRCA2*. We performed a series of *in silico, in vitro *(Figure 3) and *in vivo *(Figure 4) experiments in human breast cells. Twenty-six SNPs tagged by all five haplotypes were identified using Haploview and HapMap data. We used four criteria for selecting SNPs for further analyses. First, we selected the SNPs located inside regulatory elements described by the Ensembl database. Secondly, we selected the SNPs whose surrounding DNA sequence was predicted to potentially bind transcription factors (TF). For this purpose, 40 bps of DNA sequence surrounding these SNPs were analyzed *in silico *using TRANSFAC and MATCH for determining likelihoods of these DNA sequences to bind TFs. We used a cut-off of 0.9 for the matrix and core similarity scores; these scores correspond to the quality of a match between the DNA sequence and the TF binding matrix and the core sequence of a matrix (the five most conserved consecutive positions in a matrix), respectively. Thirdly, the TF binding site needed to overlap the SNP location. Finally, there had to be a difference between alleles in terms of presence/absence of binding and differences in scores between alleles.

**Figure 5:**
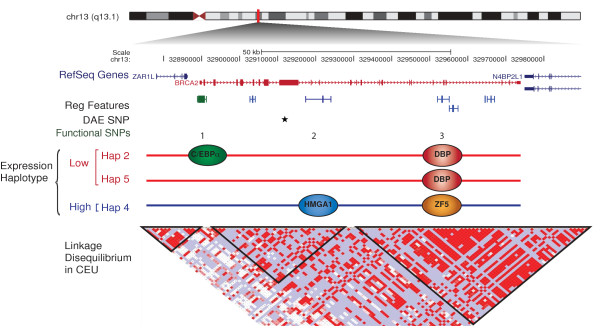
**Complex ***cis***-regulation of *BRCA2 *gene expression**. RefSeq genes mapped to the region surrounding *BRCA2*, position of regulatory features according to Ensembl, and position of DAE marker SNP rs144848 (black star) and functional SNPs (numbered). Haplotypes associated with low and high expression levels are shown with the corresponding binding of transcription factors. Linkage disequilibrium (r^2^) for CEU phased genotype data from HapMap (Data release24/phaseII). CEU, Utah residents with Northern and Western European ancestry from the CEPH collection; SNP, single nucleotide polymorphism.

The tag SNP specific for haplotype 2, rs1799943 (Functional SNP 1 in Figure 5), and another in complete LD (rs9567552) were the only SNPs localized at the promoter region of *BRCA2*. They map within a RNA polymerase II (PolII) binding site at the promoter region of *BRCA2*. To confirm binding at the promoter site we performed ChIP using RNA PolII-specific and H3K79me2-specific antibodies (a histone modification commonly associated with transcription elongation [42]) and verified occupancy at the site of these variants (Additional file 8 Figure S2). *In silico *analysis (Additional file 9 Table S6) suggested that the DNA sequence surrounding this polymorphism could bind OCT-1 with both alleles and also C/EBPα, but only in the minor A allele form. EMSAs showed that *in vitro *both alleles had the ability to bind C/EBPα Figure 3A. Next, through ChIP analysis using specific antibodies against OCT-1 and C/EBPα, we found the site to be occupied *in vivo by *C/EBPα, in two breast cancer cell lines (Figure 4A). The pull-down was significantly higher for C/EBPα in a heterozygous cell line compared to another homozygous for the G allele (Student's t test *P*-value = 0.03). No occupancy was verified for the rs9567552 site.

Three other SNPs passed our selection criteria for analysis by EMSA to evaluate binding of the predicted TFs *in vitro*, using nuclear protein extracts from two breast cancer and one normal cell line. Two of these three SNPs showed the ability to bind *in vitro *in an allelic-preferential or allele-specific manner to the predicted TFs (Figure 3B and 3C) and were further characterized by ChIP analysis.

SNP rs9567576 (Functional SNP 2 in Figure 5), tagging haplotypes 2 and 5, located in a regulatory feature in intron 11, resides within a putative binding site for the high-mobility group protein HMGA1, with the T allele better matching the consensus sequence. The band observed in the EMSA experiment (Figure 3B) was consistently stronger for the T allele in replicate experiments, corroborating the *in silico *predictions, although the G allele could still bind. In a competition EMSA experiment, an oligonucleotide centered on SNP rs9567576 competed more efficiently for binding when the SNP position was a T as compared to a G. Additionally, a consensus probe and a specific antibody against HMGA1 competed strongly for binding. The physical presence of HMGA1 at the rs9567576 site was demonstrated by site-specific ChIP analysis in both MCF-7 and MCF-10A cells (homozygous for the T allele and heterozygous at rs9567576, respectively) (Figure 4B). Although a stronger ChIP result was obtained for MCF-10A cells (genotype TG), comparative sequencing of the immunoprecipitate and the input chromatin, showed that HMGA1 binds this site in the presence of both alleles but there is a preferential binding for the T allele (Figure 4B).

SNP rs4942485 (Functional SNP 3 in Figure 5), which is tagged by rs4942440 and haplotype 4, maps inside a regulatory feature in intron 20. It was predicted that the A allele would bind the albumin D-binding protein (DBP), while the G allele could bind the zinc finger protein 161 homolog (ZF5). EMSA experiments (Figure 3C) showed strong binding for the labelled oligonucleotide containing the A allele in the form of two bands. This binding was more efficiently competed by the same unlabelled oligonucleotide than the unlabelled probe containing the G allele. Using consensus competition probes and specific antibodies for DBP and antibodies for two negative controls (OCT-1 and IgG), we confirmed that the top band corresponded to the binding of DBP. For the G allele, EMSA studies suggested potential binding of ZF5 in the form of a third lower band when in the presence of Zn^2+ ^in the binding buffer. Competition was detected when using a specific antibody against ZF5. However, stronger competition was observed in the same experiment by an antibody against DBP. These data suggest that DBP binding is stronger to the A allele of the rs4942485 sequence, but that possible binding can exist in the presence of the G allele. Our chromatin immunoprecipitation experiments in this region using specific antibodies support the *in vivo *occupancy of this site by DBP in MCF-7 cells and ZF5 in both MCF7 and MCF-10A cells (Figure 4C). Due to the low frequency of this SNP, all cell lines available for these studies were homozygous for the common A allele, preventing us from establishing allele preferences *in vivo*.

The list of the candidate SNPs for functional analysis as well as information on the gene expression levels of the transcription factors analyzed in this study, are provided as Additional files 10 and 11, respectively.

Overall, we identified three SNPs linked to haplotypes 2, 4 and 5, which are located in previously described regulatory elements, have the potential to alter the binding of TFs and are occupied *in vivo *in breast cancer and normal cell lines (Figure 5). Although rs1799943 is the tag for haplotype 2 and rs9567576 is a tag for haplotypes 2 and 5, rs4942485 tags a subset of haplotype 4 (pairwise rs4942485 versus rs4942440 r^2 ^= 0.55), which suggests that further *cis*-variants might be acting on this haplotype.

## Discussion

The aim of our study was to investigate the effect of regulatory genetic variation of the *BRCA2 *tumor suppressor gene on disease risk. To this end, we identified haplotypes associated with different expression levels of *BRCA2 *in normal breast tissue (expression haplotypes) and characterized three *cis*-acting polymorphisms that alter the binding of transcription factors at regulatory sites. We show that these expression haplotypes have an impact on the expression profile of breast cells. Also, we found some evidence of association between a high expression haplotype and lower risk of developing breast cancer among carriers of germline mutations in *BRCA2*.

The cells of individuals carrying inactivating germline mutations in the *BRCA2 *gene rely on the expression of *BRCA2 *from the remaining wild-type allele. There is evidence that these cells have a different gene expression profile and ability to repair double-strand DNA breaks compared to cells carrying two wild-type alleles [21,22]. We have shown previously that *BRCA2 *shows differential allelic expression in normal breast tissue. These observations raised the hypothesis that *cis*-regulatory variation could modify the penetrance of germline mutations in *BRCA2*, by varying the level of expression of the remaining wild-type allele, if different levels of haploinsufficiency are important. The functional effects of haploinsufficiency at a given locus may vary from tissue to tissue [22]. Many critical biological processes are context specific and the overlap of *cis*-regulatory variants across different tissues is predicted to be between 10% to 80% [43-45], suggesting that the expression haplotypes can vary from tissue to tissue. It is therefore critical to study the relevant tissue when evaluating mechanisms of effect on disease risk. The functional data that we present were obtained from the study of normal breast tissue from control individuals and breast cell lines (normal and cancer).

Haplotypes associated with different levels of gene expression were mapped in unrelated individuals, an approach that has proven powerful to identify *cis*-acting loci [46]. The difference of expression that we observed between the expression haplotypes is small, which suggests that any effect in disease will also be subtle, as expected for common variants. Further functional analysis in breast cells revealed three *cis*-acting variants affecting the binding of transcription factors which can explain at least in part the differences in expression level associated with the expression haplotypes. These *cis*-acting variants were mapped to the promoter and two intronic regulatory elements of *BRCA2*. The regulatory scenario suggested by our findings is complex, with diverse regulatory variants playing a role in different expression haplotypes. The catalogue of genetic variation is improving constantly through studies like the 1000 Genomes Project. The most recent data show more than ten predicted haplotype blocks for the region we studied here. We have analyzed a fraction of the known variation and believe that there may be more *cis*-variants regulating the expression of *BRCA2 *in breast tissue, as in others.

In a recent report, Bellacosa and colleagues have shown that *BRCA2^+/- ^*heterozygous cells have an altered gene expression profile compared with normal cells from individuals without mutations and that this effect is tissue-specific [22]. Even though the expression haplotypes we identified are associated with small differences in the expression of *BRCA2 *itself, our analyses revealed that the gene expression level of some of the same genes reported by Bellacosa *et al*. could be correlated with the expression haplotypes. These genes were, unsurprisingly, the ones with the most significant differences in the previous report. These findings suggest that even subtle changes in the expression of *BRCA2 *can have an impact on the phenotype of normal breast tissue, which can be particularly important when only one expressing allele is present.

In our association study we found some evidence that the minor allele of SNP rs4942440 (corresponding to a high expression haplotype) is associated with a protective effect for the development of breast cancer in mutation carriers. We hypothesize that the higher the expression of *BRCA2 *from the remaining wild-type allele the better the cells of a mutation carrier individual will carry out DNA repair. The evidence of association in the present study is weak. Mutation phase information for the samples used in the association study was not available. As a consequence, the group of *BRCA2 *mutation carriers who are heterozygous for this SNP consists of a mixture of individuals who have either the minor or the common allele on the wild type haplotype. We are currently collecting detailed pedigree information and will be able to use phase information in the future, which will strengthen our analysis. Genotyping of additional mutation carriers in the future will further clarify the involvement of this SNP in the penetrance of *BRCA2 *mutations in breast cancer. The selection of SNPs for the disease association analysis, as well as the gene expression studies, was performed based on our previous data associating these SNPs/haplotypes with different levels of *BRCA2 *expression. It is difficult therefore to apply an exact multiple testing, but taken together, the gene expression and association results are suggestive for the involvement of this SNP in breast cancer risk for *BRCA2 *mutation carriers.

## Conclusions

Our findings support that quite subtle levels of haploinsufficiency of a tumor suppressor gene can have biologically relevant effects. Others have reported similar effects for mutations in the *PTEN *and *TP53 *genes in mouse models and *APC *in Familial Adenomatous Polyposis in humans [47-49]. Common regulatory variation affecting the expression of *APC *and *TGFBR1 *has also been shown to contribute to susceptibility to cancer in humans [3,4]. Furthermore, common variants of the *BRCA1 *wild-type allele have also been recently suggested to modify the risk of breast cancer in *BRCA1 *mutations carriers [50]. In the future, a confirmation of these findings could be performed by direct measurement of *BRCA2 *expression levels in mutation carriers and even BRCA2 protein levels. The functional interactions between genetic variability, transcriptional regulation and cancer susceptibility are largely unexplored. Our findings highlight the importance of investigating the regulatory genetic variation of tumour suppressor genes in the search for genetic modifiers of mutation penetrance.

## Abbreviations

ANOVA: analysis of variance; bp: base pair; CEU: Utah residents with Northern and Western European ancestry from the CEPH collection; ChIP: chromatin immunoprecipitation; CI: confidence interval; DAE: differential allelic expression; EMSA: electrophoretic mobility shift assay; FCS: fetal calf serum; HR: hazard ratio; LD: linkage disequilibrium; PCR: polymerase chain reaction; SNP: single nucleotide polymorphism; TF: transcription factors.

## Conflict of Interest Statement

The authors declare that they have no conflicts of interest.

## Authors' contributions

The project was conceived by ATM and BAJP. Functional analysis was performed by ATM and MOR. Gene expression experiments were conceived, performed and analyzed by ATM, SFC, MD, CNC and CC. The study of CIMBA carriers was coordinated by GC-T. The CIMBA data management is coordinated by ACA and LM. Genotyping of CIMBA carriers was performed by GC-T and OMS. Patient DNA material and clinical data of carriers was provided by SMD, EMBRACE, DFE, SP, DF, DGE, RE, LI, JA, DE, GEMO, OMS, SM, DSL, MGV, LF, LVB, CD, HN, FJC, AKG, MAC, SWE-BRCA, RBB, KConFab, XC, JB, SH and GCT. Data were analyzed by ATM, ACA, SS, MD, CK and CNC. The manuscript was drafted by ATM, ACA and BAJP. All authors read, critically revised and approved the final manuscript.

## Supplementary Material

Additional file 1 **Table S1: Oligonucleotide sequences**. Click here for file

Additional file 2 **Table S2: List of local ethics committees that granted approval for the access and use of the data in the current study**. Click here for file

Additional file 3 **Table S3: BRCA2 mutation carriers genotyped by study for seven tag SNPs**. Click here for file

Additional file 4 **Supplementary Materials and Methods**. Click here for file

Additional file 5 **Table S4: Genotype frequencies in *BRCA2 *mutation carriers by SNP and Disease Status and Hazard-Ratio Estimate**. Click here for file

Additional file 6 **Figure S1: Study-specific estimates of per-allele hazard ratio for rs4942440**. Click here for file

Additional file 7 **Table S5: Association of breast cancer risk with common expression haplotypes**. Click here for file

Additional file 8 **Figure S2: RNA polymerase II and H3K79me2 chromatin immunoprecipitation at the promoter of *BRCA2 *and at the rs1799943 locus**. Click here for file

Additional file 9 **Table S6: TRANSFAC results**. Click here for file

Additional file 10 **Table S7: Candidate SNPs for functional analysis**. Click here for file

Additional file 11 **Figure S3: Expression of transcription factors in control normal breast tissue**. Click here for file
