# Supplementary material for: Effects of BRCA2 cis-regulation in normal breast and cancer risk amongst BRCA2 mutation carriers
Source: Breast Cancer Res. 2012 Apr 18;14(2):R63. doi: 10.1186/bcr3169 (PMC3446398; doi:10.1186/bcr3169)
Supplement: Additional file 1 — Table S1: Oligonucleotide sequences. [file bcr3169-S1.PDF]

Additional File 1 Table S1 - Oligonucleotide sequences

Method	Name	Sequence
**Sequencing**	rs11571579_seqFW	CATAGGTCTTTCCCATGTCG
	rs11571579_seqREV	TGTTGCTGCAAGGAAAACAG
	rs9567576_seqFW	ACGCACTGAAGTATGCAAGC
	rs9567576_seqREV	AATAGTCCTGGTCCCAAGCA
	rs9534174_seqFW	GCTGGGCAAATCAGTCTCTC
	rs9534174_seqREV	CTTAGAAACATGCGGCACCT
	rs4942440_seqFW	AGGCTTCAAAAAGCACTCCA
	rs4942440_seqREV	TTTCGGAAGATCCGCATAAC
	rs1799943_seqFW	AAATTTTCCAGCGCTTCTGA
	rs1799943_seqREV	TGGGTTTTTAGCAAGCATTTTT
	rs206070_seqFW	GATCCCACAATCGTGCTCTT
	rs206070_seqREV	GGTAAACCCACAAGCTTCCA
	rs4942485_seqFW	GGGGCTAAGAATGAGGGTTC
	rs4942485_seqREV	GAGCTCATCTTAAGGGTGTGG
	rs144848_seqFW	ACCACATTGGAAAGTCAATGCC
	rs144848_seqREV	CATCTGGGCTCCATTTAGACCT
**EMSA**	rs9567576-T-sense	gCATAATAATCTAAATTTACAATATCAGTATT
	rs9567576-T-antisense	gAATACTGATATTGTAAATTTAGATTATTATG
	rs9567576-G-sense	gCATAATAATCTAAATGTACAATATCAGTATT
	rs9567576-G-antisense	gAATACTGATATTGTACATTTAGATTATTATG
	rs1799943-G-sense	gGACTTATTTACCAAGCATTGGAGGAATATC
	rs1799943-G-antisense	gGATATTCCTCCAATGCTTGGTAAATAAGTC
	rs1799943-A-sense	gGACTTATTTACCAAACATTGGAGGAATATC
	rs1799943-A-antisense	gGATATTCCTCCAATGTTTGGTAAATAAGTC
	rs4942485-A-sense	gCTCAAAATAATGAGCATTCAGATATTAGCCA
	rs4942485-A-antisense	gTGGCTAATATCTGAATGCTCATTATTTTGAG
	rs4942485-G-sense	gCTCAAAATAATGAGCGTTCAGATATTAGCCA
	rs4942485-G-antisense	gTGGCTAATATCTGAACGCTCATTATTTTGAG
	CEBPALPHA-sense	gTTTGCAACAGCAGTAAAATGGGTCAAGGTT
	CEBPALPHA-antisense	gAACCTTGACCCATTTTACTGCTGTTGCAAA
	DBP-sense	gCCCATTACAAAATCATACCA
	DBP-antisense	gTGGTATGATTTTGTAATGGG
	ZF5-sense	gCAGGTACCGCGCCTTCGCTGCCA
	ZF5-antisense	gTGGCAGCGAAGGCGCGGTACCTG
	HMGA1_sense	gATTTAAGTCTAATTTAAAGT
	HMGA1_antisense	gACTTTAAATTAGACTTAAAT
**ChIP**	rs4942485_Fw	TACCCCATGGACTGTTGTGA
	rs4942485_Rev	TCACCAACTACATTACAGATGGCTA
	rs1799943_Fw	TTTGGTCTTCTGTTTTGCAGACTT
	rs1799943_Rev	TCCAATAGGCATTTTTACCTACGAT
	rs9567576_seqFW	ACGCACTGAAGTATGCAAGC
	rs9567576_seqREV	AATAGTCCTGGTCCCAAGCA
	PolII_Fw	AAGCGTGAGGGGACAGATTT
	PolII_Rev	GGACAGAGGGCTCCAGTTAG
	neg_Fw	TTGGACAGAAAATTAGTTTCC
	neg_Rev	GAAGCAAGTTGTGTTGATGAC
