# Supplementary material for: Effects of BRCA2 cis-regulation in normal breast and cancer risk amongst BRCA2 mutation carriers
Source: Breast Cancer Res. 2012 Apr 18;14(2):R63. doi: 10.1186/bcr3169 (PMC3446398; doi:10.1186/bcr3169)
Supplement: Additional file 2 — Table S2: List of local ethics committees that granted approval for the access and use of the data in the current study. [file bcr3169-S2.PDF]

Additional File 2 Table S2: List of local ethics committees that granted approval for the access and use of the data in the current study.

Study	Country	Committee approval
Epidemiological study of BRCA1 and BRCA2 mutation carriers (EMBRACE)	UK and EIRE	Anglia & Oxford MREC
Fox Chase Cancer Center (FCCC)	USA	Institutional Review Board Fox Chase Cancer Center
Genetic Modifiers of cancer risk in *BRCA1*/2 mutation carriers (GEMO)	France, USA	Comité consultatif sur le traitement de I'information en matière de recherche dans le domaine de la santé
Georgetown University (GEORGETOWN)	USA	MedStar Research Institute - Georgetown University Oncology Institutional Review Board
Pisa Breast Cancer Study (PBCS)	Italy	Azienda Ospedaliera Pisana Ethical Committee
Helsinki Breast Cancer Study (HEBCS)	Finland	Helsingin ja uudenmaan sairaanhoitopiiri (Helsinki University Central Hospital ethics committee)
Iceland Landspitali - University Hospital (ILUH)	Iceland	Vísindasiđanefnd National Boethics Committee
Kathleen Cuningham Foundation Consortium for Research into Familial Breast Cancer (KCONFAB)	Australia	Peter MacCallum Cancer Centre Ethics Committee
(KCONFAB - additional)	Australia	Queensland Institute of Medical Research - Human Research Ethics Committee
Mayo Clinic (MAYO)	USA	Mayo Clinic Institutional Review Boards
Swedish Breast Cancer Study (SWE-BRCA)	Sweden	Regionala Etikprövningsnämnden Stockholm
University of Pennsylvania (UPENN)	USA	University of Pennsylvania Institutional Review Board
