# Supplementary material for: Effects of BRCA2 cis-regulation in normal breast and cancer risk amongst BRCA2 mutation carriers
Source: Breast Cancer Res. 2012 Apr 18;14(2):R63. doi: 10.1186/bcr3169 (PMC3446398; doi:10.1186/bcr3169)
Supplement: Additional file 3 — Table S3: BRCA2 mutation carriers genotyped by study for seven tag SNPs. [file bcr3169-S3.PDF]

Additional File 3 Table S3: BRCA2 mutation carriersgenotyped by study for seven tag SNPs.

Study Group	Samples	rs1799943	rs11571579	rs9534174	rs206070	rs144848	rs4942440	rs9567576
**EMBRACE**	836	Yes	Yes	Yes	Yes	Yes	Yes	Yes
**FCCC**	54	Yes	Yes	Yes	Yes	Yes	Yes	Yes
**GEMO**	644	Yes	No	No	No	No	No	Yes
**GEORGETOWN**	16	Yes	Yes	Yes	Yes	Yes	Yes	Yes
**HEBCS**	103	Yes	No	Yes	Yes	Yes	Yes	Yes
**ILUH**	132	No	No	Yes	Yes	Yes	Yes	No
**KCONFAB**	481	Yes	Yes	Yes	Yes	Yes	Yes	Yes
**MAYO**	117	Yes	Yes	Yes	Yes	Yes	Yes	Yes
**PBCS**	56	Yes	Yes	Yes	Yes	Yes	Yes	Yes
**SWE-BRCA**	169	Yes	Yes	Yes	Yes	Yes	Yes	Yes
**UPENN**	146	Yes	Yes	No	Yes	Yes	Yes	Yes
