# Supplementary material for: Effects of BRCA2 cis-regulation in normal breast and cancer risk amongst BRCA2 mutation carriers
Source: Breast Cancer Res. 2012 Apr 18;14(2):R63. doi: 10.1186/bcr3169 (PMC3446398; doi:10.1186/bcr3169)
Supplement: Additional file 4 — Supplementary Materials and Methods. [file bcr3169-S4.DOC]

**Supplementary Methods**

*Associations with breast cancer risk for BRCA2 mutation carriers*

Associations with breast cancer risk were evaluated using women with *BRCA2* mutations from 11 studies from the CIMBA consortium for the seven SNPs defining the gene expression associated haplotypes. Sample eligibility and available information was reported in detail previously . Briefly, women were eligible if they carried pathogenic mutations in *BRCA2* and were older than 18-years at the time of recruitment. All samples included in this study were used under ethically approved protocols. The DNA samples of ten studies were genotyped by iPLEX Mass Array platform (Sequenom Inc., Newton, MA) at Queensland Institute of Medical Research, with a further study, GEMO, genotyped elsewhere by Taqman assay, using Applied Biosystems reagents (Applied Biosystems, Foster City, CA, USA), for the same seven SNPs. At least 2% of random samples were included as duplicates. Quality control criteria included over 98% of concordance between duplicates per study and over 95% call rate per study. Hardy-Weinberg equilibrium was also tested in unrelated individuals as an additional genotyping control. We also genotyped 95 DNA samples from a standard test plate (Coriell Institute) for all SNPs in order to check for consistency across the genotyping centres. Studies were excluded from the individual SNP analyses if they failed any of these criteria. Studies included per SNP in our analysis are shown in Supplementary Table 2.

Details of the statistical analysis methods for evaluating the associations between SNP genotypes and breast cancer risk have been published previously . In short, the phenotype of individuals was defined by their age at diagnosis of breast cancer or their age at last observation. For this purpose, individuals were censored at the age of the first breast cancer diagnosis (affected), ovarian cancer diagnosis (unaffected), or bilateral prophylactic mastectomy (unaffected) or the age at last observation (unaffected). In our study, mutation carriers were selected on the basis of disease status (where affected individuals are over-sampled). In view of this design, we conducted the analysis by modelling the retrospective likelihood of the observed genotypes conditional on the disease phenotypes as described previously . In this model, the breast cancer incidence was assumed to depend on the underlying SNP genotype through a Cox proportional hazards model. The effect of each SNP was modelled either as a per-allele hazard-ratio (multiplicative model) or as separate HRs for heterozygotes and homozygotes, and these were estimated on the log scale. Analyses were carried out with the pedigree-analysis software MENDEL . Heterogeneity between studies was tested by comparing the models that allowed for study-specific log-HRs against models in which the same log-HR was assumed to apply to all studies. All analyses were stratified by study group and country of residence and used calendar-year and cohort-specific breast cancer incidence rates for BRCA2 . We used a robust variance-estimation approach to allow for the non-independence among related mutation carriers .

Disease associations for the five common expression haplotypes were also explored using the software HAPSTAT , under a standard cohort analysis model and using the low expression haplotype 2 as the reference. It should be noted that this analysis does not account for the sampling design and familial relationships of the participating studies.
