# Supplementary material for: Effects of BRCA2 cis-regulation in normal breast and cancer risk amongst BRCA2 mutation carriers
Source: Breast Cancer Res. 2012 Apr 18;14(2):R63. doi: 10.1186/bcr3169 (PMC3446398; doi:10.1186/bcr3169)
Supplement: Additional file 5 — Table S4: Genotype frequencies in BRCA2 mutation carriers by SNP and Disease Status and Hazard-Ratio Estimate. [file bcr3169-S5.PDF]

Additional File 5 Table S4: Genotype frequencies in *BRCA2* mutation carriers by SNP and Disease Status and Hazard-Ratio Estimate

		Unaffected (%)	Affected (%)	HR	95% C.I.	p-value
**rs11571579**						
BRCA2	TT	461 (49.25)	496 (47.69)	1		
	CT	394 (42.09)	440 (42.31)	1.07	(0.89, 1.28)	
	CC	81 (8.65)	104 (10.00)	1.2	(0.90, 1.62)	
	2-df test					0.44
	per allele			1.09	(0.95, 1.23)	0.21
**rs144848**						
BRCA2	AA	454 (47.34)	545 (47.52)	1		
	CA	415 (43.27)	488 (42.55)	1.03	(0.86, 1.22)	
	CC	90 (9.38)	114 (9.94)	1.09	(0.82, 1.47)	
	2-df test					0.83
	per allele			1.04	(0.91, 1.18)	0.57
**rs1799943**						
BRCA2	GG	617 (55.89)	806 (54.20)	1		
	GA	411 (37.23)	561 (37.73)	1.07	(0.91, 1.26)	
	AA	76 (6.88)	120 (8.07)	1.18	(0.88, 1.57)	
	2-df test					0.49
	per allele			1.08	(0.95, 1.22)	0.24
**rs206070**						
BRCA2	GG	673 (69.96)	798 (69.63)	1		
	GA	260 (27.03)	318 (27.75)	1.08	(0.90, 1.29)	
	AA	29 (3.01)	30 (2.62)	1.06	(0.62, 1.83)	
	2-df test					0.69
	per allele			1.06	(0.90, 1.25)	0.45
**rs4942440**						
BRCA2	GG	648 (70.36)	817 (73.47)	1		
	AG	246 (26.71)	271 (24.37)	0.84	(0.70, 1.01)	
	AA	27 (2.93)	24 (2.16)	0.76	(0.43, 1.36)	
	2-df test					0.13
	per allele			0.85	(0.72, 1.00)	0.048
**rs9534174**						
BRCA2	GG	298 (32.89)	336 (31.91)	1		
	GA	429 (47.35)	512 (48.62)	1.15	(0.95, 1.39)	
	AA	179 (19.76)	205 (19.47)	1.15	(0.90, 1.46)	
	2-df test					0.34
	per allele			1.08	(0.96, 1.21)	0.22
**rs9567576**						
BRCA2	TT	519 (48.14)	688 (46.87)	1		
	GT	462 (42.86)	628 (42.78)	1.06	(0.90, 1.25)	
	GG	97 (9.00)	152 (10.35)	1.22	(0.94, 1.59)	
	2-df test					0.31
	per allele			1.09	(0.97, 1.22)	0.16
