# Supplementary material for: Effects of BRCA2 cis-regulation in normal breast and cancer risk amongst BRCA2 mutation carriers
Source: Breast Cancer Res. 2012 Apr 18;14(2):R63. doi: 10.1186/bcr3169 (PMC3446398; doi:10.1186/bcr3169)
Supplement: Additional file 6 — Figure S1: Study-specific estimates of per-allele hazard ratio for rs4942440. [file bcr3169-S6.PDF]

Additional File 6 Figure S1

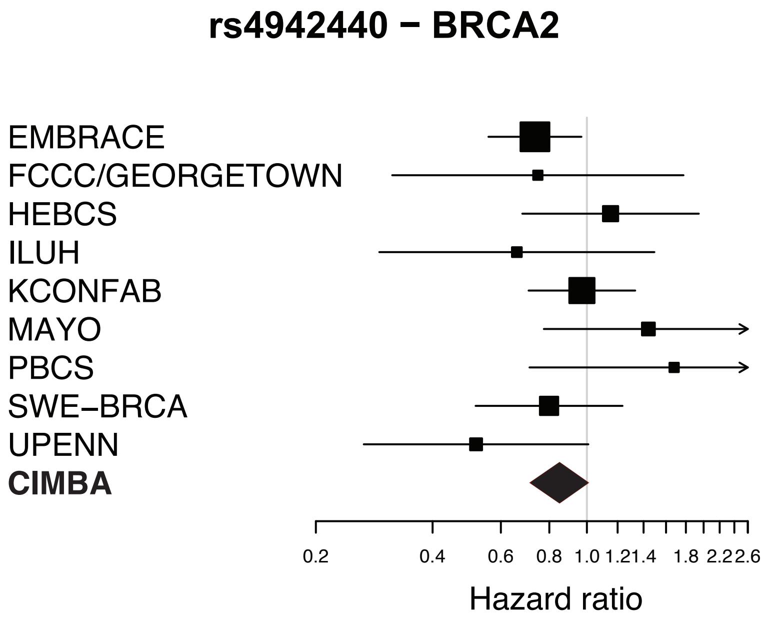

Legend: Study-specific estimates of per-allele hazard ratio for rs4942440. The area of the square is proportional to the inverse of the variance of the estimate. Horizontal lines represent the 95% confidence intervals.
