# Supplementary material for: Effects of BRCA2 cis-regulation in normal breast and cancer risk amongst BRCA2 mutation carriers
Source: Breast Cancer Res. 2012 Apr 18;14(2):R63. doi: 10.1186/bcr3169 (PMC3446398; doi:10.1186/bcr3169)
Supplement: Additional file 7 — Table S5: Association of breast cancer risk with common expression haplotypes. [file bcr3169-S7.PDF]

Additional File 7 Table S5: Association of breast cancer risk with common expression haplotypes.

Haplotype	Frequency	HR estimate	95% C.I.	p Value
2	0.2668	1 reference		
5	0.0397	1 reference		
1	0.2687	0.99	0.89-1.11	0.874
3	0.1748	0.99	0.86-1.13	0.835
**4**	**0.1571**	**0.84**	**0.73-0.97**	**0.014**
