# Supplementary material for: Effects of BRCA2 cis-regulation in normal breast and cancer risk amongst BRCA2 mutation carriers
Source: Breast Cancer Res. 2012 Apr 18;14(2):R63. doi: 10.1186/bcr3169 (PMC3446398; doi:10.1186/bcr3169)
Supplement: Additional file 8 — Figure S2: RNA polymerase II and H3K79me2 chromatin immunoprecipitation at the promoter of BRCA2 and at the rs1799943 locus. [file bcr3169-S8.PDF]

Additional File 8 Figure S2

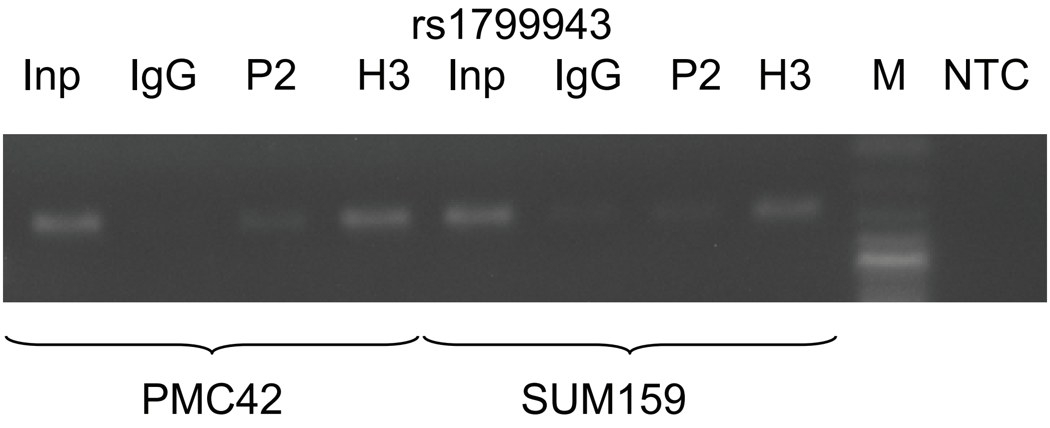

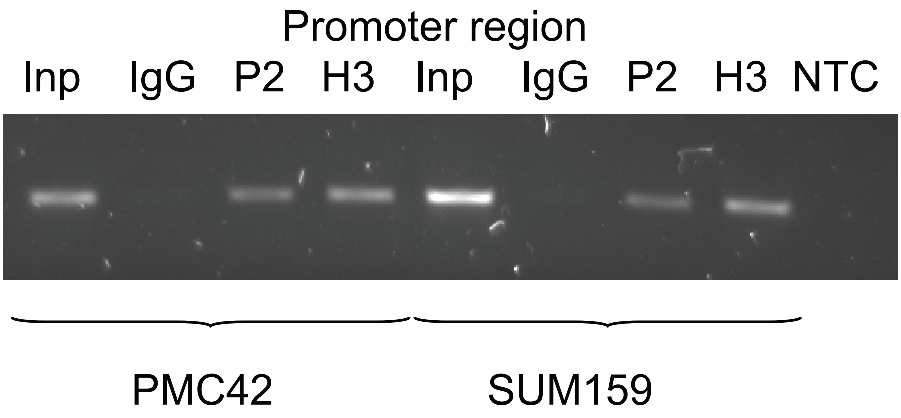

Legend: Inp - Input
IgG - IgG control antibody
P2 - PolII antibody
H3 - H3K79me2 antibody
M - Size marker
NTC - No template control
