# Supplementary material for: Effects of BRCA2 cis-regulation in normal breast and cancer risk amongst BRCA2 mutation carriers
Source: Breast Cancer Res. 2012 Apr 18;14(2):R63. doi: 10.1186/bcr3169 (PMC3446398; doi:10.1186/bcr3169)
Supplement: Additional file 9 — Table S6: TRANSFAC results. [file bcr3169-S9.PDF]

Additional File 9 Table S6: TRANSFAC results

Scanning sequence ID: rs4942485|A

matrix identifier	position	(strand)	core match	matrix match	sequence (+ strand)
V$CEBPDELTA_Q6	1	(+)	0.986	0.877	TATTTCTCAAAA
V$CEBP_Q2_01	2	(+)	0.971	0.964	ATTTCTCAAAAT
V$TCF11_01	7	(-)	0.883	0.883	TCAAAATAATGAG
V$IPF1_Q4_01	8	(-)	1	0.945	CAAAATAATGAGCaT
V$CART1_01	10	(+)	0.951	0.896	AAATAATGAGCaTTCAGA
V$CDXA_02	11	(+)	0.984	0.981	AATAATG
V$DBP_Q6	18	(+)	0.985	0.955	AGCATTC
V$PAX2_01	18	(+)	0.837	0.756	AGCaTTCAGATATTAGCCA
V$POU3F2_02	21	(-)	0.674	0.719	aTTCAGATAT
V$CEBPGAMMA_Q6	21	(-)	0.845	0.816	aTTCAGATATTAG
V$IPF1_Q4_01	23	(+)	0.914	0.872	TCAGATATTAGCCAT
V$CRX_Q4	24	(-)	0.948	0.923	CAGATATTAGCCA
V$GATA4_Q3	25	(+)	1	0.92	AGATATTAGCCA
V$YY1_Q6	33	(+)	1	0.945	GCCATCTGT

****

Scanning sequence ID: rs4942485|G

matrix identifier	position	(strand)	core match	matrix match	sequence (+ strand)
V$CEBPDELTA_Q6	1	(+)	0.986	0.877	TATTTCTCAAAA
V$CEBP_Q2_01	2	(+)	0.971	0.964	ATTTCTCAAAAT
V$TCF11_01	7	(-)	0.883	0.883	TCAAAATAATGAG
V$IPF1_Q4_01	8	(-)	1	0.954	CAAAATAATGAGCgT
V$CART1_01	10	(+)	0.951	0.81	AAATAATGAGCgTTCAGA
V$CDXA_02	11	(+)	0.984	0.981	AATAATG
V$ZF5_B	14	(+)	0.841	0.805	AATGAGCGTTCAG
V$ZF5_B	15	(-)	0.919	0.803	ATGAGCGTTCAGA
V$PAX2_01	18	(+)	0.837	0.756	AGCgTTCAGATATTAGCCA
V$CEBPGAMMA_Q6	21	(-)	0.845	0.801	gTTCAGATATTAG
V$IPF1_Q4_01	23	(+)	0.914	0.872	TCAGATATTAGCCAT
V$CRX_Q4	24	(-)	0.948	0.923	CAGATATTAGCCA
V$GATA4_Q3	25	(+)	1	0.92	AGATATTAGCCA
V$YY1_Q6	33	(+)	1	0.945	GCCATCTGT

****

Scanning sequence ID: rs9567576|T

matrix identifier	position	(strand)	core match	matrix match	sequence (+ strand)
V$POU3F2_02	1	(-)	1	0.775	ATATACATAA
V$OCT4_01	1	(-)	0.955	0.907	ATATACATAATAATC
V$PLZF_02	4	(+)	0.979	0.717	TACATAATAATCTAAATtTACAATATCAG
V$CDXA_02	6	(-)	0.984	0.944	CATAATA
V$CDP_02	7	(-)	0.685	0.783	ATAATAATCTAAATt
V$PLZF_02	7	(-)	0.979	0.803	ATAATAATCTAAATTTACAATATCAGTAT
V$CART1_01	8	(+)	0.933	0.887	TAATAATCTAAATtTACA
V$CEBPGAMMA_Q6	15	(+)	0.691	0.819	CTAAATTTACAAT
V$HMGIY_Q6	18	(-)	0.964	0.967	AATTTAC
V$CART1_01	23	(-)	0.924	0.793	ACAATATCAGTATTAACT
V$HNF1_Q6	24	(-)	1	0.877	CAATATCAGTATTAACTA
V$CDXA_02	33	(-)	1	0.937	TATTAAC

****

Scanning sequence ID: rs9567576|G

matrix identifier	position	(strand)	core match	matrix match	sequence (+ strand)
V$POU3F2_02	1	(-)	1	0.775	ATATACATAA
V$OCT4_01	1	(-)	0.955	0.907	ATATACATAATAATC
V$PLZF_02	4	(+)	0.979	0.707	TACATAATAATCTAAATgTACAATATCAG
V$CDXA_02	6	(-)	0.984	0.944	CATAATA
V$CDP_02	7	(-)	0.685	0.779	ATAATAATCTAAATg
V$CART1_01	8	(+)	0.933	0.792	TAATAATCTAAATgTACA
V$CART1_01	23	(-)	0.924	0.793	ACAATATCAGTATTAACT
V$HNF1_Q6	24	(-)	1	0.877	CAATATCAGTATTAACTA
V$CDXA_02	33	(-)	1	0.937	TATTAAC

****

Scanning sequence ID: rs1799943|G

matrix identifier	position	(strand)	core match	matrix match	sequence (+ strand)
V$CMAF_01	4	(+)	0.871	0.886	TGTTTTGCAGACTTATTTA
V$HMGIY_Q6	5	(-)	0.957	0.915	GTTTTGC
V$HAND1E47_01	6	(-)	0.871	0.838	TTTTGCAGACTTATTT
V$FXR_Q3	11	(+)	0.897	0.785	CAGACTTATTTACC
V$HNF3_Q6_01	12	(+)	0.988	0.922	AGACTTATTTACCAAGCA
V$RUSH1A_02	12	(+)	1	0.973	AGACTTATTT
V$XFD2_01	13	(-)	0.901	0.783	GACTTATTTACCAA
V$CEBPGAMMA_Q6	15	(+)	0.907	0.802	CTTATTTACCAAG
V$CEBPA_01	16	(-)	0.917	0.928	TTATTTACCAAGCA
V$SOX9_B1	25	(-)	0.912	0.827	AAGCATTGGAGGAA
V$BCL6_Q3	28	(+)	0.816	0.852	CATTGGAGGA
V$CEBPDELTA_Q6	29	(-)	0.963	0.891	ATTGGAGGAATA
V$CDPCR3_01	29	(+)	0.998	0.675	ATTGGAGGAATATCG
V$OCT1_02	31	(-)	0.992	0.851	TGGAGGAATATCGTA
V$GATA4_Q3	38	(+)	0.814	0.843	ATATCGTAGGTA

****

Scanning sequence ID: rs1799943|A

matrix identifier	position	(strand)	core match	matrix match	sequence (+ strand)
V$CMAF_01	4	(+)	0.871	0.886	TGTTTTGCAGACTTATTTA
V$HMGIY_Q6	5	(-)	0.957	0.915	GTTTTGC
V$HAND1E47_01	6	(-)	0.871	0.838	TTTTGCAGACTTATTT
V$FXR_Q3	11	(+)	0.897	0.785	CAGACTTATTTACC
V$HNF3_Q6_01	12	(+)	0.988	0.917	AGACTTATTTACCAAACA
V$RUSH1A_02	12	(+)	1	0.973	AGACTTATTT
V$XFD2_01	13	(-)	0.901	0.783	GACTTATTTACCAA
V$CEBPGAMMA_Q6	15	(+)	0.907	0.824	CTTATTTACCAAA
V$CEBPA_01	16	(-)	0.92	0.93	TTATTTACCAAACA
V$CEBPA_01	17	(+)	0.943	0.948	TATTTACCAAACAT
V$HNF3_Q6_01	17	(-)	1	0.941	TATTTACCAAACATTGGA
V$CEBP_Q2_01	19	(+)	0.977	0.97	TTTACCAAACAT
V$HNF3B_01	20	(-)	1	0.877	TTACCAAACATTGGA
V$SOX9_B1	25	(-)	0.912	0.823	AAACATTGGAGGAA
V$GATA4_Q3	27	(+)	0.791	0.848	ACATTGGAGGAA
V$BCL6_Q3	28	(+)	0.816	0.852	CATTGGAGGA
V$CEBPDELTA_Q6	29	(-)	0.963	0.891	ATTGGAGGAATA
V$CDPCR3_01	29	(+)	0.998	0.675	ATTGGAGGAATATCG
V$OCT1_02	31	(-)	0.992	0.851	TGGAGGAATATCGTA
V$GATA4_Q3	38	(+)	0.814	0.843	ATATCGTAGGTA
