# Supplementary material for: Effects of BRCA2 cis-regulation in normal breast and cancer risk amongst BRCA2 mutation carriers
Source: Breast Cancer Res. 2012 Apr 18;14(2):R63. doi: 10.1186/bcr3169 (PMC3446398; doi:10.1186/bcr3169)
Supplement: Additional file 10 — Table S7: Candidate SNPs for functional analysis. [file bcr3169-S10.PDF]

Additional File 10 Table S7 - Candidate SNPs for functional analysis

Haplotype	Tag SNP	Candidate SNPs							
2,5	rs11571579, rs9567576	rs11571579	rs1801406	rs3752451	rs9567576	rs9567578	rs11571662	rs1029304	
1	rs144848	rs144848							
1,3	rs9534174	rs206079	rs9534174						
4	rs4942440	rs1853521	rs2126042	rs2320236	rs4942439	rs4942440	rs4942443	rs4942448	rs4942485
3	rs206070	rs206070	rs206077	rs206081	rs206118	rs206120	rs543304		
2	rs1799943	rs9567552	rs1799943						
