# Supplementary figures and images for: Effects of BRCA2 cis-regulation in normal breast and cancer risk amongst BRCA2 mutation carriers
Source: Breast Cancer Res. 2012 Apr 18;14(2):R63. doi: 10.1186/bcr3169 (PMC3446398; doi:10.1186/bcr3169)

# Expression of Transcription Factors in Control Normal Breast Tissue (n=33)

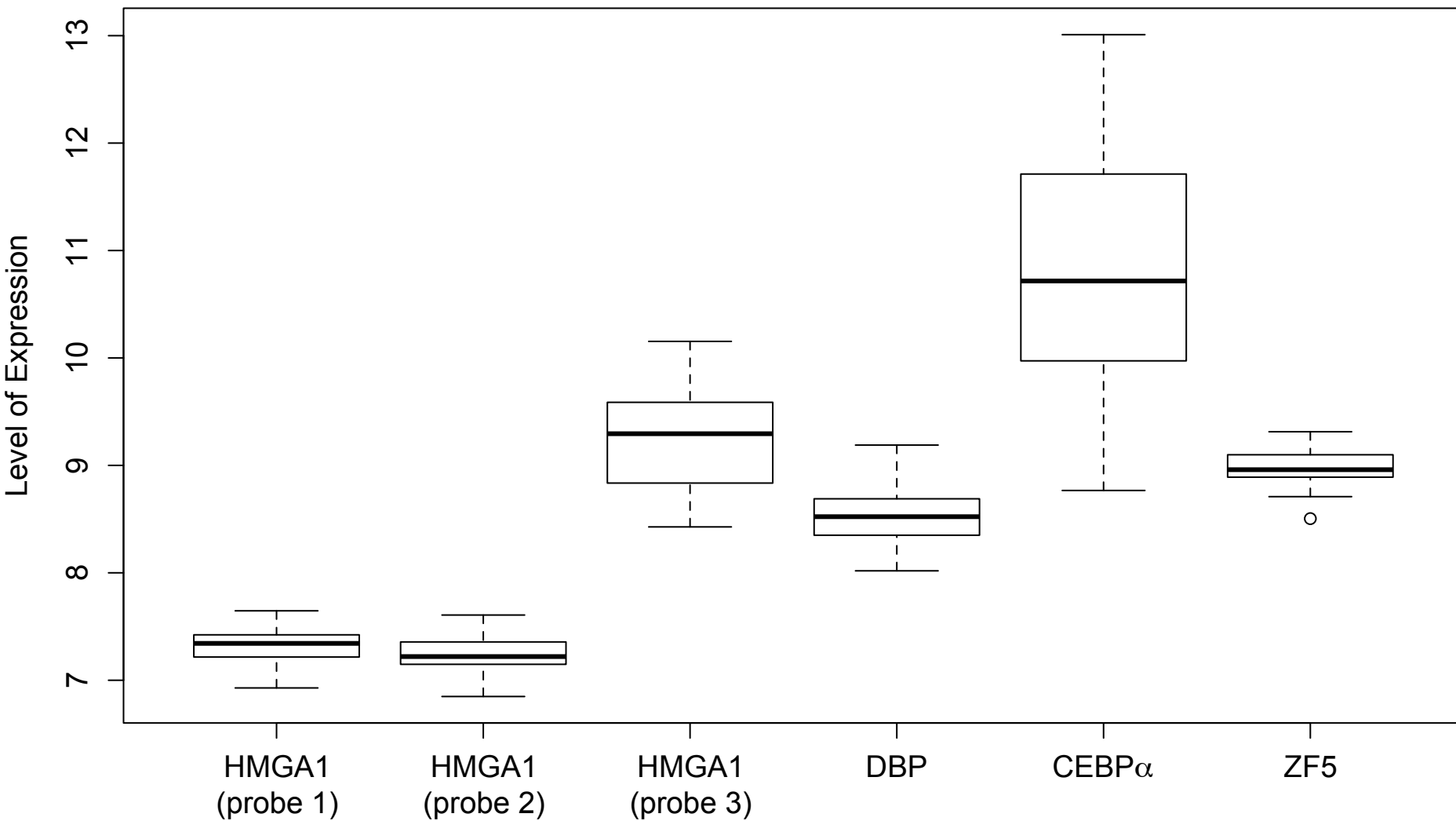

Supplement: Additional file 11 — Figure S3: Expression of transcription factors in control normal breast tissue. [file bcr3169-S11.PDF]
